# Less frequent follow-up in routine care than in trials does not impact resistance selection in patients failing DRV/r or ATV/r first line treatment

**DOI:** 10.7448/IAS.17.4.19744

**Published:** 2014-11-02

**Authors:** Anne-Genevieve Marcelin, Charlotte Charpentier, Marc Wirden, Diane Descamps, Vincent Calvez

**Affiliations:** 1 Virology, Pitie-Salpetriere Hospital, Paris, France; 2 Virology, Bichat-Claude Bernard Hospital, Paris, France

## Abstract

**Introduction:**

Selection of resistance mutations on antiretroviral therapy (ART) including darunavir (DRV/r) or atazanavir (ATV/r) has been reported infrequently but mainly in clinical trials where patients were followed very frequently (at least four to five clinical visits and viral load measurements per year). The aim of this study was to evaluate the rate of resistance at failure and mutational patterns emerging in patients receiving DRV/r or ATV/r based-regimen as first line treatment and followed in standard clinical practice with less clinical visits and viral load measurements (median=2 per year).

**Methods:**

We studied 1,518 patients starting their first line antiretroviral therapy and followed during at least two years (n=799 TVD+DRV/r, n=70 KVX+DRV/r, n=618 TVD+ATV/r, n=31 KVX+ATV/r). The median viral load at baseline was 76,000 copies/mL and the median CD4 cell count 384 cell/mm^3^. Virological failure was defined as two consecutive viral load=50 copies/mL after previous suppression <50 copies/mL, or failure to achieve <50 copies/mL. Predicted susceptibility was determined using the last ANRS algorithm.

**Results:**

Among the 1,518 patients, 193 (12.7%) failed during the two years of follow-up. Among patients failing TVD+DRV/r (n=95), the emerging mutations observed were RT M184V (n=8; 8%) and Pro V32I (n=1; 1%). Among patients failing KVX+DRV/r (n=8), the emerging mutations observed were RT M184V (n=3; 37%) and Pro I47V (n=1; 12%). Among patients failing TVD+ATV/r (n=86), the emerging mutations observed were RT M184V (n=9; 10%), Pro N88S (n=2; 2%) and Pro I50L (n=1; 1%). Among patients failing KVX+ATV/r (n=4), the emerging mutations observed were RT M184V (n=2; 50%) and no Pro mutation. Most of patients retained virus predicted to be susceptible to all antiretrovirals (22 virus became resistant to 3TC/FTC and three became resistant to ATV). None of them became resistant to DRV.

**Conclusions:**

Among 1,518 patients in routine care who started their first line treatment with DRV/r or ATV/r, very few of them (1.4%) selected resistance mutations at failure with three patients selecting an ATV resistant virus. None of them became resistant to DRV. The less frequent follow-up of patients in routine care compared to clinical trials does not impact the resistance selection rate in patients treated by boosted DRV or ATV based regimen.

